# Next-Generation Protein–Ligand Interaction Networks: APEX as a Powerful Technology

**DOI:** 10.3390/proteomes13030026

**Published:** 2025-06-23

**Authors:** José Miguel Quintero-Ferrer, Lucas Silva de Oliveira, Paula Marian Vieira Goulart, Thiago Albuquerque Souza Campos, Coralie Martin, Philippe Grellier, Izabela Marques Dourado Bastos, Sébastien Charneau

**Affiliations:** 1 Laboratory of Biochemistry and Protein Chemistry, Department of Cell Biology, Institute of Biological Sciences, University of Brasilia, Brasilia 70910-900, Brazil; jochemiguel22@gmail.com (J.M.Q.-F.); lucassoliveira@outlook.fr (L.S.d.O.); paulamvgoulart@gmail.com (P.M.V.G.); thiagocampos0301@hotmail.com (T.A.S.C.); 2 UMR 7245 MCAM Molecules of Communication and Adaptation of Microorganisms, Muséum National d’Histoire Naturelle, CNRS, 75231 Cedex 05 Paris, France; coralie.martin@mnhn.fr (C.M.); philippe.grellier@mnhn.fr (P.G.); 3 Pathogen-Host Interface Laboratory, Department of Cell Biology, Institute of Biological Sciences, University of Brasília, Brasilia 70910-900, Brazil; dourado@unb.br

**Keywords:** protein–protein interactions, proximity labeling, molecular networks, protein interactomes, engineered ascorbate peroxidase, mass spectrometry

## Abstract

Peroxidases are essential enzymes that catalyze redox reactions, with wide-ranging biological implications. Among these, an enhanced ascorbate peroxidase (APEX) has emerged as a valuable tool for studying intricate intracellular events with spatiotemporal precision, particularly in protein–protein, protein–RNA, and protein–DNA interaction networks in living cells. This review discusses APEX’s structural and functional attributes, its evolution through genetic engineering, and its transformative applications in high-resolution mapping used for proteomic and transcriptomic studies. Furthermore, it highlights recent advancements in substrate innovation and addresses current challenges and future directions in leveraging APEX for cutting-edge biological research.

## 1. Introduction

Protein–ligand interactions, which form the basis of diverse biomolecular networks, are central to deciphering the complex mechanisms that regulate cellular functions. The biological activity of proteins is intrinsically linked to their ability to interact with other molecules, including an antibody with an antigen [1], a protein receptor with a ligand [2], a transport protein with a ligand [3], an enzyme with a substrate [4], and cytoskeleton meshwork proteins with mechanical properties [5]. The ligands could be inorganic or organic compounds, including vitamins [6], hormones [7], nucleic acids [8], carbohydrates [9], lipids [10], and proteins [11]. These interactions regulate critical pathways, such as signal transduction, gene expression, metabolic processes, cell differentiation, and cellular adaptation to environmental changes, making them essential targets for scientific and therapeutic exploration.

Despite their importance, studying these complex interactions in living cells poses significant challenges due to the dynamic, transient, and spatially restricted nature of biomolecular interactions. Therefore, advanced tools are required to enable the precision, versatility, and high-resolution capabilities necessary to map protein–ligand interactions effectively. Among these tools, L-ascorbate peroxidase (APX, EC 1.11.1.11) is a Class I cytosolic heme peroxidase that remains catalytically active under reducing conditions. Naturally expressed in plants, chlorophytes, red algae, and certain protists, APX plays a central role in detoxifying excess hydrogen peroxide. This enzyme catalyzes the reduction of hydrogen peroxide to water using ascorbate as an electron donor. This enzymatic activity is crucial for maintaining redox homeostasis and limiting oxidative damage, thereby underscoring its essential role in hydrogen peroxide regulation within plant cells [12,13].

Enhanced variants of APX (APEX) have further improved the labeling specificity, efficiency, and cellular targeting, enabling the detailed mapping of biomolecular interactions across subcellular compartments [14,15]. The utility of APEX extends beyond proteomics, offering significant advantages in mapping specific cell compartments and studying protein interactomes, cellular structures, and subcellular processes in living cells. These advancements underscore APEX’s versatility and potential to drive deeper insights into molecular mechanisms. This review provides a comprehensive analysis of APEX technology, detailing its structural and functional attributes, engineering advancements, and applications in proteomic and transcriptomic studies. Additionally, we explore the latest innovations in APEX-based labeling strategies, addressing current limitations and potential future directions.

## 2. Peroxidase

Peroxidases are a diverse class of enzymes involved in redox reactions, which mediate the electron transfer between substrates. Their primary role involves the reduction of hydrogen peroxide, an electron acceptor, through the oxidation of various organic and inorganic substrates. These enzymes are ubiquitous across all five kingdoms of life, playing essential roles in processes such as cellular signaling, the defense against oxidative stress, and the metabolism of reactive oxygen species [16]. Peroxidases are generally classified into two main groups: heme-containing peroxidases and non-heme-containing peroxidases [17].

Heme-containing peroxidases are characterized by the presence of a heme b group, an organometallic compound that serves as a prosthetic group in these enzymes. The heme group is a coordination complex containing an iron (III) ion bound to protoporphyrin IX, which facilitates electron transfer during substrate oxidation and supports efficient redox activity. A key structural feature of these enzymes is the conserved histidine residue that acts as the proximal ligand to the iron atom, along with conserved arginine and histidine residues functioning as distal ligands [18,19]. This arrangement is essential for both catalytic efficiency and substrate specificity.

Non-animal heme-containing peroxidases are categorized into three distinct classes, each with specialized functions. Class I, the most divergent, primarily neutralizes excess hydrogen peroxide [20]. Class II consists of fungal manganese and lignin peroxidases, which play a key role in lignin degradation [21]. Class III, the largest group, includes plant secretory peroxidases involved in the oxidation of lignin, auxin, and secondary metabolites [22].

The functional versatility of peroxidases is exemplified by horseradish peroxidase (HRP), one of the most extensively studied members of this enzyme family, which is a glycoprotein incorporating a heme group and two calcium ions, both critical for its structural stability and catalytic activity [23,24]. In molecular biology, HRP is invaluable for signal detection and amplification, catalyzing the oxidation of chromogenic and chemiluminescent substrates to generate measurable signals in assays such as the ELISA, Western blotting, and immunohistochemistry [25,26].

The catalytic adaptability of HRP extends to the oxidation of a broad spectrum of phenolic substrates, making it a valuable tool for protein labeling and conjugation strategies [27]. This versatility arises from its flexible active site architecture, which accommodates structurally diverse compounds. This broad substrate specificity has driven research aimed at improving HRP’s stability and activity under non-native conditions, including the engineering of HRP variants to enhance its substrate specificity, thermal stability, and resistance to inactivation. These efforts aim to optimize HRP for applications in synthetic biology, diagnostics, and industrial processes.

## 3. APX: A Robust Intracellular Peroxidase

A significant limitation of HRP is its inactivation within the mammalian cytosol, primarily due to the reducing environment [12]. This environment disrupts the formation of disulfide bonds and interferes with calcium-binding sites, both essential for HRP’s structural stability and catalytic function. Moreover, the limited cytosolic availability of calcium further exacerbates these challenges, severely compromising the HRP activity and restricting its utility in intracellular studies, such as protein interaction analysis and cellular process exploration [28].

To address these limitations, APX has emerged as a superior alternative. Unlike HRP, APX is inherently adapted to intracellular conditions, maintaining stability and catalytic efficiency in the absence of disulfide bonds or calcium ions [12]. This unique characteristic allows APX to function effectively in environments where HRP is rendered inactive, establishing it as an indispensable tool for investigating intracellular processes.

APX, a heme-containing Class I peroxidase, regulates intracellular hydrogen peroxide levels by catalyzing the oxidation of L-ascorbate (ascorbic acid), which is predominantly synthesized via the D-mannose/L-galactose pathway in plants [29]. This activity is essential for maintaining redox homeostasis, thereby enhancing cellular defense mechanisms in plants, algae, and certain cyanobacteria [18,30,31]. Owing to its versatile chemistry, ascorbate functions as a free radical scavenger and a reductant for iron and copper [32,33]

Beyond its role in redox regulation, APX also modulates oxidative stress-responsive signaling pathways, enabling plants to adapt to a variety of environmental challenges. In higher plants, APX isoenzymes are distributed across multiple cellular compartments, differing in both their subcellular localization and amino acid sequences. They are found in the stroma and thylakoid membranes of chloroplasts, as well as in the membranes of glyoxysomes, peroxisomes, and the cytosol. These characteristics underscore the critical role of APX in safeguarding cellular integrity, maintaining redox homeostasis across distinct organelles, and facilitating adaptive responses under stress conditions [34,35,36,37,38,39,40].

The cytosolic form of APX typically exists as a dimer composed of identical subunits, each with a molecular mass of approximately 28 kDa, which is approximately 40% smaller than HRP [12]. While this dimeric configuration exhibits a high peroxidase activity, isoforms localized to chloroplasts or mitochondria may assemble into different oligomeric states, including homodimer and complexes [41,42,43]. Notably, APX demonstrates a catalytic activity across a broad temperature range of 20–37 °C and at a pH of 7.0 [44,45]. This range is significant as it closely aligns with the optimal temperature for many enzymes commonly found in warm-blooded organisms.

Structurally, APX distinguishes itself from HRP by the absence of disulfide bonds, signal peptides, and glycosylation, features commonly associated with the stability and targeting of other peroxidases. Furthermore, APX’s independence from calcium ions for catalytic activity enhances its adaptability to diverse physiological conditions, including those characterized by calcium deficiency or reducing environments [46,47]. These features suggest an evolutionary advantage, enabling APX to maintain an efficient intracellular trafficking and function in varied cellular contexts.

## 4. Catalytic Mechanism of APX

The successful expression of APX enabled a large-scale production, facilitating purification and crystallographic studies that unveiled complex details of its three-dimensional structure and catalytic mechanisms [18,47]. A defining feature of APX is its dual substrate-binding capability: one site interacts with its physiological substrate, ascorbic acid, while the other binds non-physiological aromatic compounds [48,49].

The primary site, near the γ-edge of the heme, binds L-ascorbate via specific interactions with Arg172, Lys30, and the heme 6-propionate. A second spatially separate site accommodates aromatic substrates, likely near the δ-meso carbon, as indicated by the structural data with salicylhydroxamic acid. Notably, mutations at Arg172 or Lys30 impair ascorbate oxidation but do not affect the activity toward aromatic compounds, confirming that these sites are structurally independent and functionally specialized [50].

**Figure 1:**
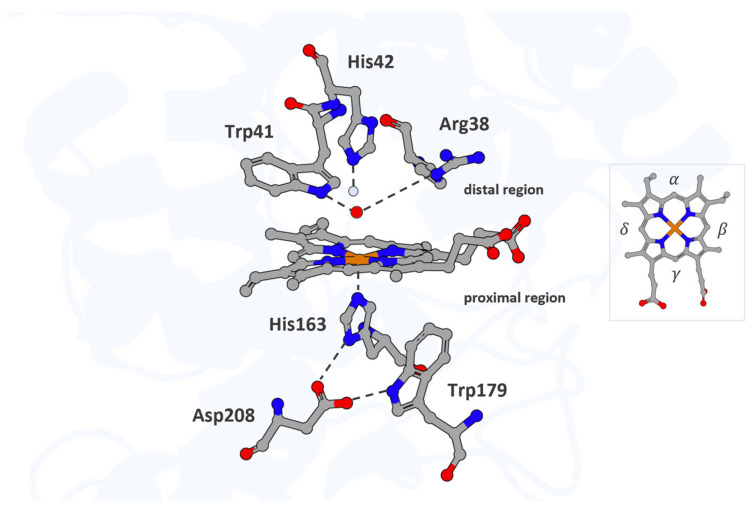
The active site of APX. The heme cofactor and the residues His163, Trp179, and Asp208 in the proximal region, along with His42, Trp41, and Arg38 in the distal region, are shown as atom-colored sticks (iron, carbon, nitrogen, and oxygen colored in orange, gray, blue, and red, respectively). Waters are represented in the light blue sphere, and ferryl oxygen occupies the distal pocket and coordinates the heme iron. Hydrogen bonds are indicated by dotted lines. The heme periphery is annotated to indicate the α-, β-, γ-, and δ-edges. Structural mapping is based on the Protein Data Bank entry 1OAG (PDB_00001OAG).

The active site of APX and its immediate environment are characterized by conserved structural features typical of the peroxidase superfamily. In all structurally characterized peroxidases, the proximal His163 and Asp208 adopt conserved positions, forming the canonical Asp–His–Fe triad, in which the heme iron is coordinated by His163, whose non-coordinating Nδ atom hydrogen bonds to a neighboring aspartate. This Asp–His–Fe triad is a defining structural motif of the peroxidase superfamily and has traditionally been considered indispensable for catalysis [18,49]. By contrast, a proximal Trp179 is unique to cytochrome c peroxidase (CcP) and APX, whereas most other peroxidases possess phenylalanine at this position. In APX, on the distal face, the iron ligand is flanked by a hydrogen-bonding Trp41, alongside His42 and Arg38, which interact with the oxo group via bridging water molecules and contribute to its stabilization during catalysis [49,51] (Figure 1).

**Figure 2:**
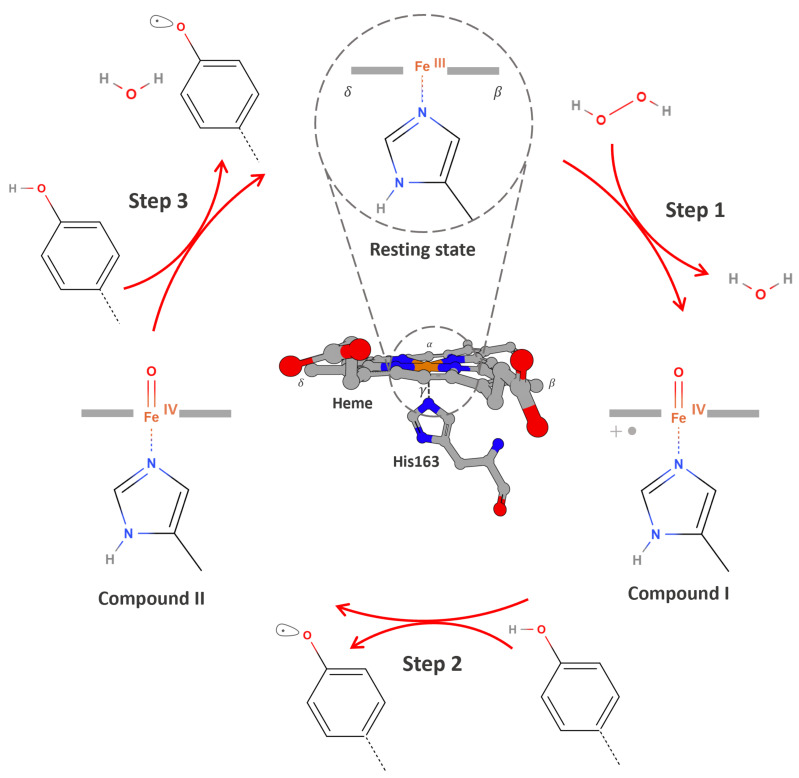
An overview of the catalytic cycle of APX. Step 1: The resting ferric enzyme Fe (III) reacts with hydrogen peroxide to generate Compound I, a high-valent iron–oxo intermediate consisting of an Fe (IV) = O center and a porphyrin-centered radical Fe (IV) = O/Por^•^⁺. Step 2: A phenolic substrate (e.g., guaiacol, biotin-tyramide) binds near the solvent-exposed δ-edge of the heme and donates one electron and one proton to Compound I. This results in the formation of a phenoxyl radical and Compound II Fe (IV) = O, which lacks the porphyrin radical. The substrate radical is then released into the surrounding environment. Step 3: A second equivalent of the phenolic substrate undergoes the same process, donating an electron and proton to reduce Compound II, generating a second phenoxyl radical and regenerating the enzyme in its resting Fe (III) state, thereby completing the catalytic cycle.

The catalytic mechanism of APX involves a conserved sequence of redox transitions mediated through transient, high-valent iron–oxo intermediates that drive the stepwise oxidation of electron-donating substrates. In the catalytic cycle, the first intermediate form is Compound I, a highly oxidized species resulting from the two-electron, two-proton transfer from hydrogen peroxide to the ferric Fe (III) heme ion [49,52,53]. Structurally, unlike in other peroxidases where a tryptophan residue is oxidized to form a cationic radical, the oxidation in APX occurs at the δ-edge of the heme macrocycle, resulting in a ferryl heme Fe (IV)=O coupled to a porphyrin π-cation radical (Por^•^⁺), more precisely described as Fe (IV)–L^•^. (Figure 2, Step 1). Compound I acts as the primary oxidant, initiating electron transfer from the substrate to the enzyme’s active site [14,52,53,54,55].

The one-electron reduction of Compound I by a substrate yields Compound II, which retains the ferryl heme moiety Fe (IV)=O but lacks the porphyrin-centered radical (Figure 2, Step 2). Compound II is reduced back to the resting state via a second electron transfer from a substrate (Figure 2, Step 3) [52,54,56,57,58].

Compound III is another intermediate that can form in some peroxidases, particularly under excess hydrogen peroxide, and is frequently associated with enzymatic inactivation, “suicide inactivation”, as evidenced by kinetic studies; however, detailed structural or mechanistic information in APX has not yet been reported [14].

Kinetic assays using guaiacol as a model substrate demonstrated its oxidation into tetraguaiacol, enabling precise measurements of the initial velocity, catalytic turnover, and substrate affinity [14,50]. Nonetheless, two inhibitory mechanisms were identified: a reversible inhibition at moderately high hydrogen peroxide levels and an irreversible inactivation accumulating over prolonged catalytic cycles [12,14]. These findings underscore limitations in long-term stability, despite the optimal activity under standard conditions.

Beyond their catalytic role, certain isoforms exhibit a dual substrate specificity, oxidizing both ascorbate and glutathione. This versatility enhances plants’ resilience to abiotic stressors, such as heat and salinity, by broadening the antioxidant capacity [59].

## 5. Engineering APX

The identification of the first cDNA encoding APX by Mittler and Zilinskas marked a decisive advance in the study of this enzyme family [60]. This breakthrough enabled the cloning and expression of multiple APX genes, providing the basis for comprehensive structural and functional analyses. Early heterologous expression studies of cytosolic APX isoenzymes from peas and soybeans in *E. coli* provided their catalytic properties and physiological roles in plants [47,61,62,63]. These initial studies laid the foundation for subsequent biochemical characterizations, enabling systematic comparisons across isoforms and species.

An unexpected discovery arose with the isolation of APX enzymes from bovine eyes [64]. This finding revealed that APX functions are not confined to plants, significantly broadening their research scope and suggesting a role in mammalian physiology, where it may contribute to the oxidative stress regulation across diverse biological systems.

**Figure 3:**
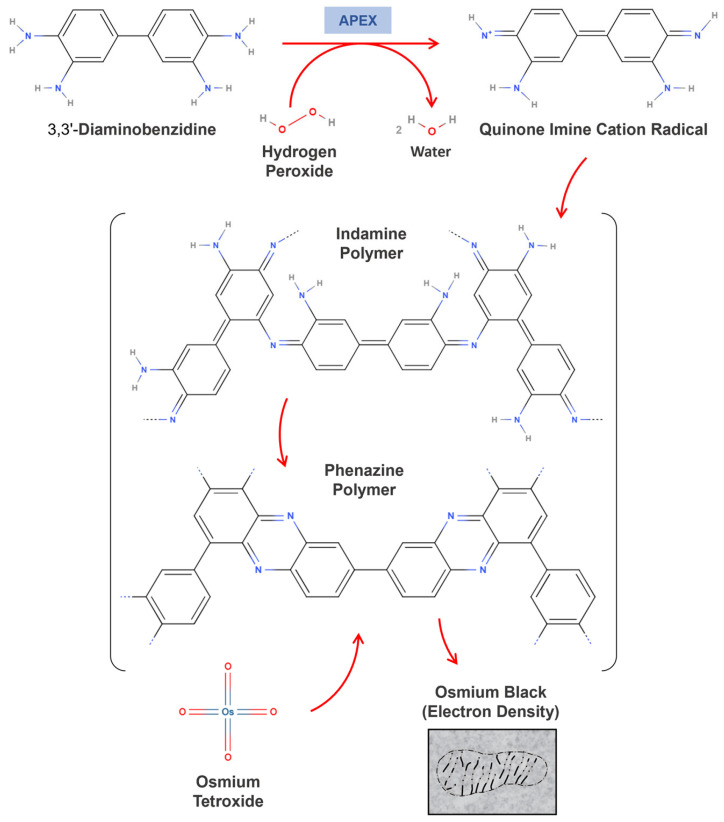
The enzyme-catalyzed DAB polymerization mechanism. The figure presents a hypothetical mechanism for the polymerization of 3,3’-Diaminobenzidine catalyzed by the iron porphyrin enzyme, leading to the formation of indamine or phenazine coupling products. These products contain primary aromatic amino groups, enabling them to react with osmium tetroxide. Additionally, the indamine polymer may undergo cyclization to form the phenazine polymer.

In 2012, Alice Ting’s group introduced a genetically engineered version of APX, derived from *Glycine max*, as a reporter for electron microscopy (EM) applications [12]. This innovative approach engineered APEX (E for engineered) to catalyze the oxidation of 3,3’-Diaminobenzidine (DAB) in the presence of hydrogen peroxide, initiating a polymerization reaction that produces indamine or phenazine products that form an insoluble brown precipitate. These products, upon forming complexes with osmium tetroxide, enable high-resolution imaging with an exceptional contrast (Figure 3) [65,66,67,68,69].

In mammalian cells, the utility of APEX is demonstrated through its fusion to target proteins, followed by a fixation with glutaraldehyde and incubation with diaminobenzidine (DAB) and hydrogen peroxide, triggering the rapid polymerization of DAB, enabling a high-resolution visualization while preserving native cellular structures. Unlike conventional EM contrast methods, which often rely on light activation or antibody-based tagging and can introduce significant structural artifacts, APEX operates under mild labeling conditions. Its enzymatic specificity and the localized nature of DAB polymerization minimize perturbations to the cellular architecture, maintaining structural integrity close to the physiological state. Furthermore, the Ting group extended APEX’s versatility by utilizing it for biotin-tyramide mediated protein proximity labeling in live cells [70].

The evolutionary trajectory of APEX variants—from the wild-type enzyme to the engineered forms APEX, APEX2, and APEX3—exemplifies the power of structural and functional optimization in enzyme engineering for biological applications. The cytosolic form of wild-type APX assembles into a noncovalent homodimer stabilized by ionic interactions, as seen in pea cytosolic APX structures [18]. While this dimerization is integral to its physiological function, it significantly impairs the enzyme’s stability and utility in proximity labeling experiments under cellular conditions.

**Figure 4:**
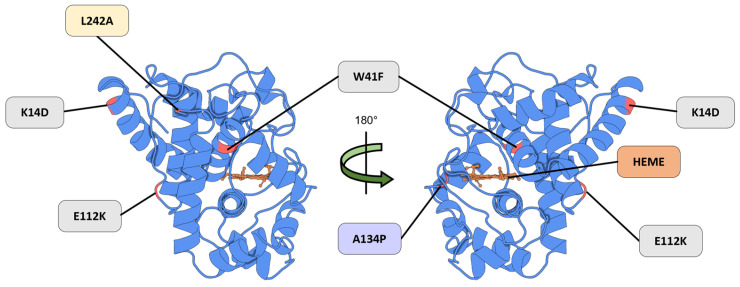
A representation of the APEX mutation strategy. The K14D and E112K mutations were introduced into APEX to reduce dimerization, while the W41F mutation restored catalytic activity (Gray). The APEX2 variant incorporates an additional A134P substitution (Purple), which confers an enhanced catalytic efficiency. The APEX3 variant includes the L242A mutation (Yellow), resulting in an improved nuclear localization relative to APEX2. The heme cofactor (Orange) is required for redox activity. Structural mapping is based on the Protein Data Bank entry 5L86 (PDB_00005L86).

These limitations were addressed through directed evolution and rational mutagenesis, leading to the development of APEX. Engineered via a yeast display evolution, monomeric APEX retained the core α-helical fold while incorporating key mutations—K14D and E112K, which reduced dimerization, and W41F, which restored the catalytic activity (Figure 4, highlighted in gray) [12,71]. Further improvements were achieved with the introduction of the A134P mutation, enhancing the catalytic efficiency, thermal stability, heme-binding affinity, and hydrogen peroxide tolerance in the optimized variant APEX2 (Figure 4, highlighted in purple) [14].

Structural studies of APEX2 suggest that specific mutations optimize the heme microenvironment and enhance substrate accessibility, markedly improving the enzymatic performance in complex cellular contexts. Critically, APEX2 functions as a monomer, which is an essential feature for maintaining the proper folding and activity within the reducing conditions of the cytosol, where dimeric variants often misfold or become inactive. Furthermore, the overexpression of dimeric APEX can lead to organelle aggregation, particularly in mitochondria and the endoplasmic reticulum, thereby disrupting the cellular architecture. The engineered monomeric configuration circumvents these issues, supporting a high catalytic activity even at low expression levels while minimizing cytotoxicity [72,73].

Building upon these optimizations, the substitution of the canonical axial histidine with a genetically encoded Nδ-methyl histidine (NMH) in APEX2 enhanced the resistance to irreversible inactivation while preserving catalytic efficiency. Notably, despite the absence of the conserved hydrogen-bonding interaction traditionally deemed essential for activity, APEX2-NMH exhibited a five-fold higher total turnover number while maintaining a comparable or slightly improved catalytic performance [74].

Nonetheless, early variants of APEX2 displayed a cytoplasmic localization bias, primarily due to hydrophobic residues within the nuclear export signal (NES) regions. Although substitutions within these regions reduced this bias, they also compromised labeling efficiency.

To overcome this trade-off, the APEX2-L242A mutant—designated APEX3—was developed (Figure 4, highlighted in yellow) [75]. This variant successfully retained the peroxide-dependent labeling activity while minimizing the cytoplasmic retention. APEX3 integrates the catalytic advantages of APEX2 while eliminating cytoplasm-biased localization, positioning it as a powerful tool for applications requiring a spatially confined proteomic analysis within the nucleus [75].

The choice of an APEX variant is guided by the experimental objectives and methodological requirements of each study. APEX2 was engineered to enhance the catalytic performance, particularly by reducing the sensitivity to hydrogen peroxide. APEX3 was subsequently optimized for whole-cell distribution, avoiding cytoplasm-biased localization and extending its applicability to studies of the nuclear proteome. Therefore, the selection of an appropriate APEX variant depends on the specific biological context and research question, with APEX2 offering an improved catalytic efficiency and APEX3 enabling effective nuclear targeting.

To further expand APEX versatility, split-APEX technology was introduced. This innovation divided the enzyme into inactive N-terminal and C-terminal fragments that regained peroxidase activity upon their recombination during molecular interactions, with a focus on the specific and precise targeting of protein–protein interactions, RNA–protein interactions, and organelle contact sites. Split-APEX has been applied to mammalian cell membranes, noncoding RNA scaffolds, and mitochondria-associated endoplasmic reticulum contact sites [76,77]. Additional advancements, such as the development of a cysteine-free mutant, further stabilized APEX2-tagged proteins, optimizing their performance in diverse experimental contexts [78].

## 6. High-Resolution Mapping with APEX

The versatility of APEX arises from its ability to generate free radicals through the oxidation of diverse aromatic substrates, a process that can be chemically modulated to enhance reactivity and specificity. Notably, APEX oxidizes substrates as varied as DAB for electron microscopy (EM) contrasts, guaiacol for colorimetric assays, Amplex™ Red for fluorometric detection, and a variety of biotins and other substrates (Table 1). Interestingly, APEX catalyzes the oxidation of aromatic compounds—traditionally processed by Class III peroxidases—at rates comparable to ascorbic acid oxidation [46,79,80]. The substrate promiscuity of APEX can be attributed to its wide binding pocket and high oxidation potential of 1.16 V [52], which allows it to effectively catalyze the oxidation of a diverse range of substrates. These features are fundamental for biochemical assays and cellular labeling applications.

**Figure 5:**
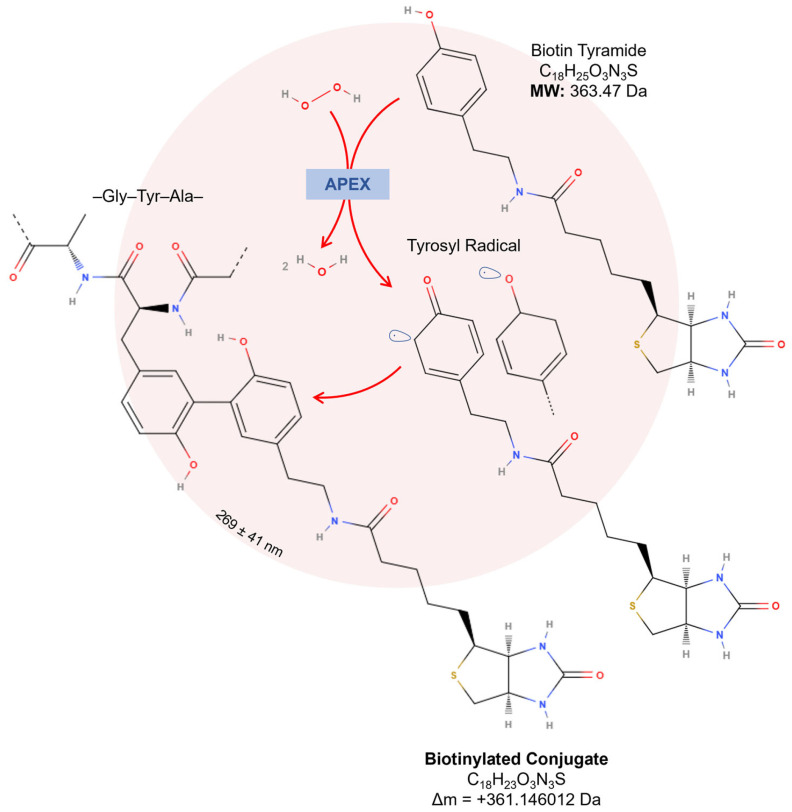
Proximity-dependent labeling using engineered APEX. In the presence of hydrogen peroxide, APEX oxidizes two biotin-tyramide/biotin–phenols, generating highly reactive biotin–phenoxyl radicals that covalently bind to nearby electron-rich amino acids, such as tyrosine, tryptophan, and cysteine. With a limited diffusion of 269 ± 41 nm, biotinylation occurs in a confined region, improving the spatial resolution and reducing nonspecific labeling.

Concerning proteome applications, engineered APEX mediates the oxidation of the most common substrate, biotin-tyramide (also known as biotin–phenol), in the presence of hydrogen peroxide. This reaction generates biotin–phenoxyl radicals, which covalently bind to electron-rich amino acids, including tyrosine, tryptophan, and cysteine [12,70]. These radicals exhibit a high reactivity, a short lifespan of <1 ms, and a limited tagging radius, initially estimated at approximately 20 nm [70]; however, recent evidence indicates a broader diffusion radius of 269 ± 41 nm [91]. Proteins labeled through this approach are purified using streptavidin-conjugated resin and identified by a liquid chromatography tandem mass spectrometry analysis (LC-MS/MS) [14,92]. This technique is advantageous because it bypasses dependencies on protein–protein interactions or organelle integrity post-labeling, enabling the precise mapping of protein interactomes (Figure 5).

This approach is particularly relevant in the context of proteoforms, as an alternative translation initiation and other post-translational modifications can generate isoforms with distinct subcellular localizations and functional properties [93]. Notably, the mislocalization of specific proteoforms has been implicated in various pathologies. In Alzheimer’s disease, extracellular deposits of amyloid-β and intraneuronal accumulations of tau proteoforms are defining features, whereas the Lewy pathology of Parkinson’s disease is characterized by intracellular inclusions of α-synuclein [94]. These findings underscore the critical need to map the distribution of proteoforms within cellular compartments, unravel some of the partner proteins, and thus infer different proteoform functionalities, a task that can only be achieved using high-precision proximity-dependent labeling techniques such as APEX.

**Figure 6:**
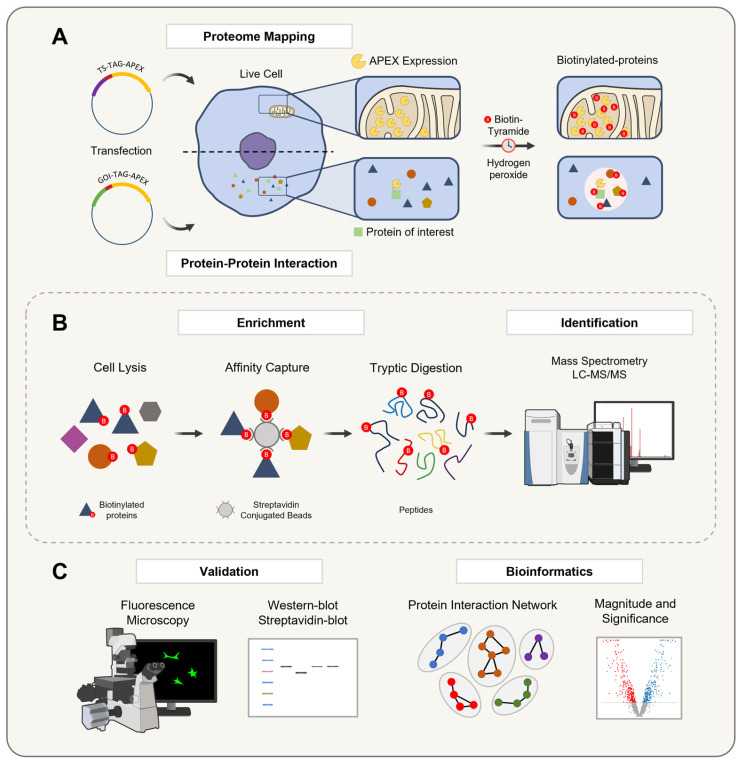
A schematic representation of the APEX technology workflow for organelle proteome mapping and protein–protein interaction studies. The workflow for APEX-mediated proximity labeling involves two primary approaches: (**A**) Targeting APEX to a specific organelle via a targeting sequence (TS) or fusing it to a gene of interest (GOI) to express a protein of interest. In the presence of hydrogen peroxide, APEX catalyzes the oxidation of biotin-tyramide into a short-lived biotin–phenoxyl radical, which mainly biotinylates tyrosine residues on nearby proteins in a proximity-dependent manner, reflecting the localization of the bait protein. (**B**) The biotinylated proteins are then enriched and purified by an affinity capture with a streptavidin pulldown assay, digested into peptides, and identified by LC-MS/MS to generate a comprehensive proximal proteome profile. (**C**) Finally, the biotinylation process is validated in living cells using Western blotting and fluorescence microscopy, followed by a specific data analysis to visualize and interpret results based on the experimental design, ensuring the robust identification and validation of protein–protein interactions within their native cellular context.

The APEX-based proximity labeling technology has been introduced into proteomics in two main approaches: for organelle/structure proteome mapping [15,70,95,96,97] or for identifying protein–protein interactions [98,99,100,101,102] (Figure 6). In the first approach, APEX is fused to a targeting sequence that directs the enzyme to specific subcellular compartments. Once localized, APEX catalyzes the biotinylation of nearby proteins, enabling the detailed characterization of organelle proteomes. This method provides valuable insights into the composition and biological functions of these compartments.

In the second approach, APEX is fused to a protein of interest, where it remains active whether fused at the N-terminus or C-terminus [71]. This fusion facilitates the labeling and identification of interacting partners, supporting the construction of protein interaction networks. By mapping these interactions, researchers can advance their understanding of dynamic cellular processes and their underlying mechanisms.

Both approaches—episomal expression and genomic integration—can be employed to introduce APEX into cells. In episomal expression, APEX is delivered via plasmids, enabling a transient or stable expression without altering the genome [70]. In contrast, genomic integration, achieved through gene editing techniques, ensures a stable and long-term expression of APEX [102]. While episomal expression allows for flexible and rapid protein production, genomic integration provides more consistent expression levels, making it preferable for long-term studies. Once the APEX expression is established, its labeling capabilities facilitate advanced proteomic analyses, including both bottom-up and top-down approaches.

Following transfection and APEX expression, proteins within the cells undergo biotinylation during the culture. These biotinylated proteins are then purified using streptavidin affinity chromatography. To confirm the labeling efficiency and proper APEX localization, a validation is performed through Western blotting and immunofluorescence microscopy. Finally, the biotinylated proteins are identified and analyzed by mass spectrometry, enabling a comprehensive exploration of organelle proteomes and protein interaction networks [14,78].

The versatility of APEX has enabled its broad application across diverse cellular and organismal systems, allowing for the detailed spatial and functional characterization of proteomes in various biological contexts. Enhanced APEX protocols have been instrumental in profiling proteomes across diverse subcellular compartments and cellular structures, including the mitochondria [15,70,103,104], primary cilia [105], endoplasmic reticulum [104,106], stress granules [107], and cellular dynamics signaling complexes [108,109]. Other applications include mapping glycoconjugate-binding proteins [110,111], ER-plasma membrane junctions [112], mitochondrial nucleoids [113], neuronal synapses [114], G-protein-coupled receptors [108,109], nuclear and nucleolar proteins [115], DNA damage response dynamics [115], histone modifications [116], and protein topology and localization in the mitochondrial matrix [86,117], endoplasmic reticulum [118], and extracellular vesicle exosome [72].

In this context, APEX-based strategies have also proven to be effective for determining the membrane protein orientation. CryoAPEX, which combines chemical fixation with high-pressure freezing and APEX tagging, enables the precise localization of membrane proteins while preserving the subcellular membrane architecture [106,119,120]. This method is compatible with electron tomography, facilitating the reconstruction of high-resolution three-dimensional maps of membrane proteins, such as the human FIC (filamentation induced by cAMP) protein and the endoplasmic reticulum single-pass membrane protein HYPE.

The versatility of APEX has enabled its integration into a wide array of experimental systems for subcellular proteomic profiling. Its robustness and ability to function in living cells have supported applications across diverse model organisms and pathogens. In human cells, APEX has been employed to map subcellular proteomes and investigate protein–protein interactions under physiological conditions [118]. In *Drosophila melanogaster* and *Caenorhabditis elegans*, it has facilitated the analysis of tissue-specific protein networks during development [44,121,122]. In parasitic protozoa, such as *Plasmodium falciparum*, APEX technology has advanced the characterization of specialized organelles—such as rhoptries—while in *Toxoplasma gondii*, it has been used to investigate mechanisms that inhibit necroptosis, a process crucial for long-term parasite persistence within tissue cysts during a chronic infection. These insights contribute to a deeper understanding of host–pathogen interactions. [102,123]. Similarly, in *Trypanosoma brucei*, this strategy has been used to elucidate the flagellar proteome [97]. In prokaryotic systems, such as *Mycobacterium tuberculosis*, APEX has enabled the identification of compartmentalized bacterial proteins relevant to virulence [124]. Additionally, in the yeast *Saccharomyces cerevisiae*, it has proven to be effective for studying organelle-specific proteomes and dynamic protein localization during stress responses [96,125].

Beyond these established systems, APEX technology holds significant promise for expanding proximity labeling into other models. In *Danio rerio*, a transparent vertebrate model widely used in developmental biology, it could enable a high-resolution, cell-type-specific proteomic profiling during organogenesis and neurodevelopment [126]. In intracellular bacterial pathogens, such as *Listeria monocytogenes* and *Salmonella enterica* [127,128], APEX-based labeling may help reveal how these microbes modulate host cell proteomes, shedding light on mechanisms of bacterial virulence and host manipulation. Collectively, these diverse and emerging applications underscore the adaptability of this technology for dissecting complex biological processes across multiple experimental contexts.

### Biotinylation as Post-Translational Modification (PTM)

**Figure 7:**
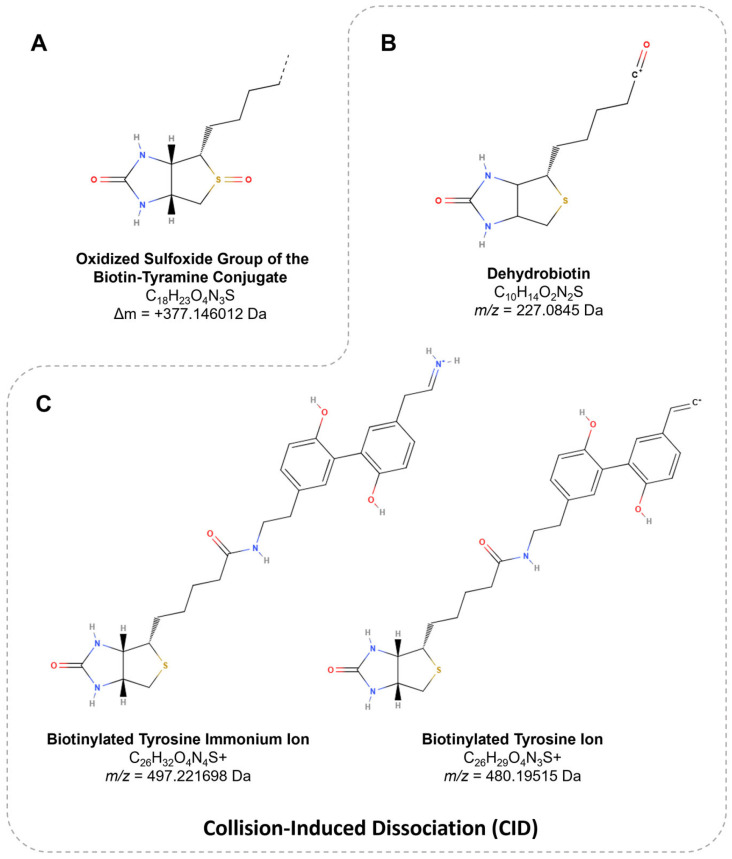
The structure of biotin derivatives. (**A**) The variation in the oxidized sulfoxide group of biotin-tyramide, resulting in a mass shift of +377.146012 Da. (**B**) The *m*/*z* value of 227.0845 corresponds to dehydrobiotin, a product ion specific to biotin. (**C**) The *m*/*z* values of 497.221698 (immonium ion) and 480.19515 (immonium-related ion) represent characteristic ions associated with biotinylated tyrosine.

Concerning biotin-tyramide, the main substrate used for APEX-based proteomic workflows, optimizing APEX labeling hinges on the accurate identification and thorough characterization of biotinylation sites, as this directly influences the precision and efficiency of downstream analyses. Tyrosine represents the predominant site of biotinylation, accounting for 98.5% of modifications, while minimal labeling is detected at other residues, such as tryptophan (0.5%) and cysteine (1%). In a mass spectrometry analysis, this biotinylation event corresponds to a mass shift of +361.146012 Da (Figure 5) [117]. However, the oxidation of the thioether group in biotin-tyramide probes can decrease the peptide signal intensity and complicate the identification of low-abundance or inefficiently labeled proteins. The oxidized peptide variant, with a delta mass of +377.146012 Da (Figure 7A), may exhibit up to ~20% of the signal intensity of non-oxidized peptides in human cells [86].

Peptides harboring PTMs, such as biotinylation, produce characteristic fragment ions upon collision-induced dissociation (CID), including dehydrobiotin at *m*/*z* 227.0845 (Figure 7B), identified in biotin-tyramide [129] and Sulfo-NHS-LC-Biotin conjugates [130]. Additionally, biotinylated peptides generate ions uniquely associated with biotinylated tyrosine, such as the immonium ion at *m*/*z* 497.221698 and a related ion at *m*/*z* 480.19515 (Figure 7C). The presence of these ions collectively contributes to an 11–12% increase in the peptide detection sensitivity [117,131].

To further enhance the utility of these findings, the data have been integrated into proteomic analysis software, such as PatternLab V, which aids researchers in the biological interpretation of APEX-based experiments [132,133]. Alternatively, fragmented ions can be manually analyzed regardless of the software used [102]. Overall, these features of biotin-tyramide as a PTM should be considered when using APEX technology, which facilitate peptide identification, improve PTM site localization accuracy, and support the optimization of analytical strategies [134]. Databases such as Unimod [135], a comprehensive repository of protein modifications for mass spectrometry, provide experimentally validated mass values based on elemental compositions. These data allow for the precise determination of mass shifts arising from both natural and artificial modifications, thereby facilitating a more accurate interpretation of mass spectrometry results.

## 7. APEX Technology to Study DNA and RNA

**Figure 8:**
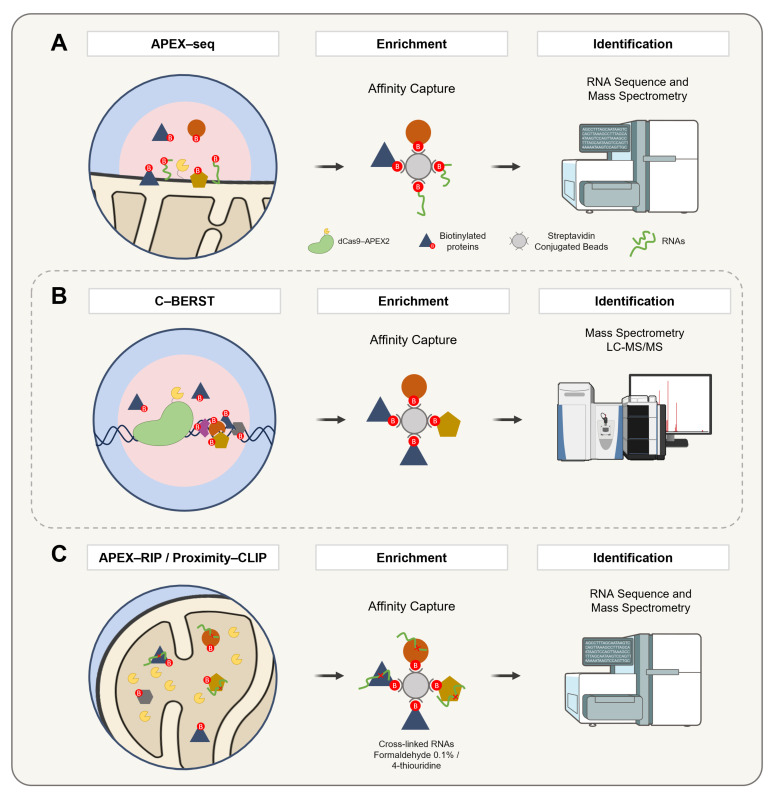
A schematic representation of APEX2-based methodologies for the spatially resolved identification of protein, DNA, and RNA interactomes. (**A**) APEX-seq enables spatial RNA mapping by targeting APEX2 to specific subcellular compartments, where biotin–phenol and hydrogen peroxide induce the selective biotinylation of nearby RNAs. These are captured with streptavidin beads, enriched via poly(A) selection, and analyzed by RNA sequencing. (**B**) C-BERST employs a fusion construct comprising catalytically inactive Cas9 (dCas9) and APEX2. The complex is guided to specific genomic loci (e.g., telomeres or centromeres) via single-guide RNAs. Upon the addition of biotin–phenol and hydrogen peroxide, APEX2 catalyzes the generation of biotin–phenoxyl radicals that covalently label proteins in the vicinity. The biotinylated proteins are subsequently enriched via a streptavidin affinity purification and identified by mass spectrometry. (**C**) APEX-RIP and Proximity-CLIP combine proximity biotinylation with RNA capture for transcriptome profiling. In APEX-RIP, cells expressing compartment-targeted APEX2 are exposed to biotin–phenol and hydrogen peroxide, followed by cross-linking with 0.1% formaldehyde to stabilize protein–RNA interactions. Biotinylated complexes are purified by the streptavidin pulldown, and co-purified RNAs are analyzed by qRT-PCR or RNA-seq. Proximity-CLIP further enhances the spatial and molecular resolution by incorporating the 4-thiouridine labeling of nascent RNAs. After APEX2 labeling in the presence of biotin–phenol and hydrogen peroxide, cells are subjected to UV irradiation (λ > 312 nm) to induce covalent cross-linking between proteins and RNAs. Lysates are then processed for a streptavidin-based enrichment, and associated RNAs are identified by RNA-seq, providing high-resolution maps of localized ribonucleoprotein complexes.

Beyond its applications in proteomic labeling, APEX has emerged as a versatile tool for targeting diverse biomolecules, including RNA and DNA. A pivotal development in this field was the introduction of APEX-seq in 2019 by Fazal and colleagues (Figure 8A). This technique leverages the reactivity of biotin-tyramide radicals with RNA guanosine residues to enable subcellular transcriptome profiling. By employing APEX2, biotinylated RNAs are isolated through a streptavidin-based purification, facilitating the capture of RNAs proximal to specific proteins [136,137]. Subsequent innovations, such as the development of biotin-tyramide derivatives including biotin-aniline and biotin-naphthylamine (Table 1), have further enhanced the efficiency of RNA and DNA labeling, respectively [88]. These advancements have significantly expanded APEX’s utility, enabling more precise and detailed subcellular explorations.

Recent innovations in APEX substrates have further enhanced their capabilities. For instance, alkyne–phenol has emerged as a novel substrate, demonstrating a 94% specificity and improved labeling efficiency in yeast mitochondria compared to traditional biotin-tyramide [90]. Similarly, biotin-aniline has enabled the characterization of the mitochondrial matrix transcriptome and confirmed the presence of glycan-conjugated RNA on cell surfaces [138]. These substrate advancements have refined the precision and versatility of APEX, paving the way for more detailed investigations into molecular interactions and cellular dynamics.

Extending the application of APEX to the study of DNA-associated proteomes, the dCas9-APEX2 system has been adapted to map DNA-associated proteomes through restricted spatial tagging, a method termed the chromatin-based enzymatic reporter for specific tagging (C-BERST) (Figure 8B). In initial applications, telomeres and centromeres were targeted, enabling the specific profiling of their subnuclear proteomes [139]. In parallel, an alternative strategy known as genomic locus proteomics (GLoPro) was introduced to investigate non-repetitive single loci [140]. This approach involved the design and expression of five distinct sgRNAs tiling the same genomic region across separate HEK293T cell lines. By integrating the resulting datasets, shared background signals were effectively filtered out, thereby enhancing the locus specificity. To mitigate artifacts arising from the constitutive expression of dCas9-APEX2, an inducible promoter system was employed to finely regulate expression levels.

A novel APEX2-based proximity labeling strategy was developed to map protein interactions with mitochondrial DNA G-quadruplexes in living cells, overcoming the limitations of traditional approaches lacking subcellular specificity. This method enabled the identification of several mtDNA G4-binding proteins, including DHX30, a previously uncharacterized helicase shown to localize to both the cytoplasm and mitochondria. DHX30 resolves mtDNA G4 structures, and its activity was found to reduce the glycolytic metabolism in tumor cells—an effect reversed by the G4 stabilizer RHPS4 [141].

Building on these breakthroughs, APEX-RIP (RNA Immunoprecipitation) (Figure 8C) integrates the proximity biotinylation by APEX with RNA Immunoprecipitation to directly capture and identify RNAs within distinct subcellular compartments. This method employs formaldehyde cross-linking to preserve RNA localization patterns, offering an exceptional specificity and sensitivity. As a result, APEX-RIP overcomes the limitations of traditional fractionation methods, providing a robust platform for studying RNA dynamics [142].

In a complementary approach, Proximity-CLIP (Figure 8C) integrates APEX-mediated biotinylation with UV-induced cross-linking in the presence of 4-thiouridine (4SU) to enable the profiling of RNA-binding proteins and their associated RNAs. This technique maps regulatory interactions within critical cellular compartments, such as the nucleus, cytoplasm, and cell junctions, offering a comprehensive view of RNA–protein interactions [143,144].

Further expanding the scope of APEX applications, a study introduced two innovative strategies, applying APEX proximity labeling using complementary approaches to target human telomerase RNA (hTR). The first approach involved conjugating hTR to the bacteriophage MS2 RNA stem–loop, which specifically binds to an MS2 coat protein-fused APEX2 (MCP-APEX2). The second approach utilized a catalytically inactive Cas13-APEX2 fusion (dCas13-APEX2), programmed by a guide RNA (gRNA) to target unmodified hTR. These methods enabled the tagging of endogenous proteins in the vicinity of specific RNAs for a subsequent identification by mass spectrometry. When applied to hTR, this approach successfully identified known interaction partners as well as unexpected hits, including an enzyme responsible for RNA post-transcriptional modifications that influence telomerase activity [145]. Building on this strategy, a subsequent study employed dCas13d-APEX2 to map cell cycle-dependent interactors of the FAS1 mRNA, which encodes the β subunit of fatty acid synthase in yeast. This analysis revealed that the glycolytic enzyme Tdh3p binds to FAS1 mRNA and is essential for the periodic expression of the Fas1p protein, thereby uncovering a direct link between metabolic regulation and the RNA-mediated control of gene expression [146]. Altogether, these studies collectively demonstrate the versatility of APEX in addressing diverse biological questions, from mapping RNA–protein interactions to visualizing RNA dynamics in situ. These developments highlight APEX’s potential for studying the spatial and functional dynamics of the transcriptome and proteome in living cells.

Expanding methodological capabilities, the combination of APEX with the organic–aqueous phase separation of cross-linked protein–RNA complexes (APEX-PS) enables the selective enrichment of RNA-binding proteins from specific subcellular compartments. This approach revealed novel proteins localized to the nucleus, nucleolus, and outer mitochondrial membrane [147].

**Figure 9:**
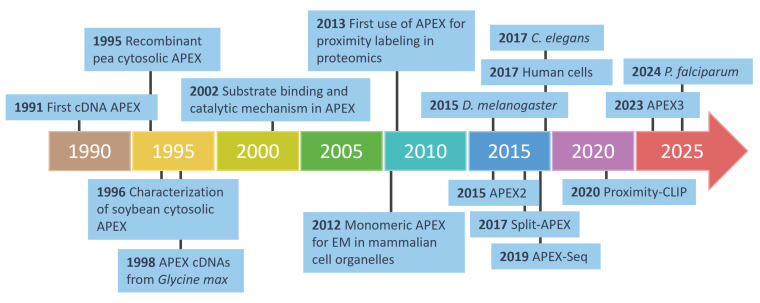
The development of APEX technology in proteomics and biological applications. This timeline highlights key milestones in the development of APEX technology, from its initial discovery in 1991 to its evolution into advanced tools such as APEX2, APEX3, and split-APEX. Over the years, APEX has been adapted for diverse applications, including electron microscopy (EM), proximity labeling, and proteomics, across various model organisms such as *C. elegans*, *D. melanogaster*, *P. falciparum*, and human cells. Innovations like APEX-Seq further demonstrate their versatility in mapping RNA interactions and cellular structures with a high precision.

The advancements in APEX-based proximity labeling for studying protein–ligand interactions are summarized in Figure 9, while Table 2 presents the diverse methodologies enabled by APEX technology in proteomics and genomics.

## 8. Advantages and Challenges

The remarkable diversity of proteoforms contributes to the profound complexity of cellular proteomes [149], supports intricate molecular interaction networks [150], and presents significant analytical challenges [151]. In this context, APEX offers significant advantages over conventional protein interaction methods, including yeast two-hybrid (Y2H) [152] and co-immunoprecipitation (Co-IP) [153]. Unlike these conventional techniques, APEX operates in situ, preserving interactions within their native cellular environment [154]. While Y2H relies on protein activity reconstitution in yeast, which may not reflect native conditions and Co-IP is prone to protein complex dissociation during lysis, APEX overcomes these limitations by enabling a real-time interaction detection in living cells [15]. This in situ capability significantly enhances the reliability of APEX for studying dynamic cellular processes [14].

APEX also excels in spatial resolution by reducing the risk of diffusion beyond the target labeling area. Notably, APEX is particularly advantageous in bacterial systems, as it does not depend on cytoplasmic metabolites like ATP, which are absent in the bacterial periplasm [155]. This feature enhances its applicability in diverse biological contexts.

For the application of the APEX system in the study of transient or weak protein–protein interactions, combining APEX with chemical cross-linking agents, such as formaldehyde, enables the stabilization of ephemeral interactions prior to biotinylation. This dual strategy facilitates the capture of short-lived complexes that might otherwise dissociate during the labeling process [156]. Alternatively, fusing APEX to compartment-specific nanobodies restricts the biotinylation to targeted subcellular regions, thereby enhancing the spatial precision of the labeling [157]. Moreover, a notable strength of APEX is its compatibility with correlative light–electron microscopy (CLEM) [158], allowing the integration of molecular interaction data with the ultrastructural context. This integration provides a more comprehensive understanding of cellular processes by bridging molecular and structural biology.

Lastly, APEX’s compatibility with advanced imaging modalities, including electron microscopy, underscores its utility in high-resolution studies [159]. The enzyme’s ability to generate free radicals that covalently bind to proximal proteins ensures a high specificity and spatial precision, which are critical for detailed proteomic mapping. These attributes establish APEX as a cornerstone technology in cellular and molecular biology, driving innovation and discovery in the field.

One of the greatest challenges in APEX technology is the elution of biotinylated proteins/peptides. The interaction between biotin and streptavidin is extremely strong (K_d_ ~10^−15^ M) [129,160], which complicates the enrichment of biotinylated proteins/peptides [15]. Factors such as the use of excessive streptavidin-coated beads or insufficient washing steps can lead to nonspecific binding, reducing the efficiency of capturing true APEX-tagged proteins. To mitigate this, it is recommended to determine the minimal bead amount through titration and apply stringent washing conditions to eliminate non-biotinylated proteins [15]. A recent report suggests that using 80–100 µL of the bead volume is sufficient to enrich up to 2 mg of protein input [161].

Moreover, even using stringent washes with low amounts of urea and detergents, followed by an elution with the Laemmli buffer at high temperatures to recover biotinylated proteins, does not seem sufficient to remove efficiently non-biotinylated interactors [15,70,86,102,112], resulting in a significant background of non-biotinylated proteins often remaining in the proteomic data. Consequently, many studies rely on quantitative proteomic approaches to determine the sub-compartment proteomes [70,108,162,163] or APEX-tagged protein [89,112,148,164], often based on a ratiometric analysis without considering biotinylation sites.

Beyond APEX, other proximity labeling methods based on the biotin ligase from *E. coli* have been developed, including BioID (and its enhanced version BioID2) [163,165,166], DiQ-BioID (dimerization-induced quantitative BioID) [167], TurboID [89,163,168], AirID (ancestral BirA for proximity-dependent biotin identification) [169], and split-AirID [170]. While these methods also provide biotinylation, they often suffer from slow enzymatic kinetics, requiring long incubation periods with biotin (6–24 h for BioID and ~10 min for TurboID) [165,166,168]. In contrast, APEX enables efficient protein labeling within one minute of the hydrogen peroxide exposure, minimizing the background associated with an extended incubation and allowing for the precise capture of highly dynamic protein interactions [14]. This rapid reaction makes APEX the optimal choice for capturing rapid or transient interactions with an unparalleled temporal resolution.

The ratiometric analysis, while useful for determining protein localization within specific compartments or complexes, is limited in providing topological information about transmembrane proteins [86,163]. Common approaches to enrich biotinylated proteins, such as trypsin digestion followed by streptavidin-coated bead enrichment [86,160,171] or on-bead digestion [86,108,162], have proven to be inefficient in some cases.

To address these limitations, researchers have developed alternative strategies to enhance the recovery of biotinylated proteins identified by mass spectrometry. One approach involves the use of anti-biotin antibodies to enrich biotinylated peptides after tryptic digestion [117,160]. While this method is more efficient than traditional avidin–biotin interactions, it is technically demanding and often yields low recovery rates [163]. Other challenges include improving the accuracy of protein localization, particularly for proteins with dual cellular locations (e.g., inner vs. outer mitochondrial membrane) [15,86,118] and addressing issues related to the probe permeability and accessibility of residues in macromolecular complexes [15,172].

Biotin-tyramide, the main probe used for APEX assays, has a limited membrane permeability [15,89,90,96,172]. To overcome this, alternative probes such as desthiobiotin-phenol (DBP) have been developed, offering an improved membrane permeability [86,163]. Additionally, the development of the Spot-ID method based on the use of DBP, which focuses on the comprehensive identification of labeled sites, has shown promise in increasing the accuracy of the biotinylated site identification and reducing false positives [86,163]. Regarding the elution of biotinylated peptides obtained through Spot-ID, organic MS-friendly solvents, such as high-concentration formamide, appear to enhance the recovery efficiency [86,163]. Similarly, alkyne–phenol has been shown to be an equally effective and specific substrate for APEX, yielding a higher number of biotinylated site hits identified by MS [89,90,172]. Although comparable in efficiency to the Spot-ID method, alkyne–phenol is significantly more expensive.

Beyond probe optimization, recent advances in enzyme engineering have opened new avenues for enhancing the catalytic performance and stability of peroxidases used in proximity labeling. Gutierrez-Rus and colleagues [173] demonstrated that the integration of a Nuclear Magnetic Resonance-based hotspot identification with computational protein design platforms, such as FuncLib, can predict mutations that enhance the enzymatic function and thermal stability, by analyzing chemical shift perturbations upon substrate- or transition-state analog binding. Subsequently, FuncLib-guided mutagenesis enables the optimization of both the catalytic efficiency and structural robustness while minimizing deleterious trade-offs. Molecular dynamics simulations further assist by revealing non-productive substrate conformations that can be eliminated through rational engineering. This integrated strategy has already proven to be successful in improving the catalytic performance of Kemp eliminase, achieving enzymes with triple the activity and enhanced stability. Applying such an approach to APEX variants could refine substrate interactions and optimize kinetic parameters, expanding their utility in diverse biological contexts.

Choosing the best approach for enriching biotinylated proteins/peptides depends on the biological model, financial resources, and laboratory infrastructure. While APEX technology excels in capturing short-lived events and offers a variety of membrane-permeable probes, each experimental model presents unique challenges. Therefore, a careful experimental design is essential to avoid setbacks and ensure reliable results. APEX continues to be a powerful tool for high-resolution cellular and molecular research, driving innovation in the study of protein–protein interactions and cellular dynamics.

## 9. Conclusions

The APEX system has emerged as a powerful tool for elucidating protein interaction networks within native cellular contexts. A key advantage of this technique is its ability to label proteins in living cells while preserving molecular complexes and maintaining cellular compartment integrity. Thus, APEX technology enables us to study the subcellular localization of proteoforms and their different partners that they interact with in more detail and consequently deduce different functions among the proteoforms.

APEX has become an essential method for studying protein–ligand interactions under physiological conditions, preserving spatial associations while providing a high-resolution mapping of interaction networks. This unparalleled precision not only enhances our understanding of molecular mechanisms and their cellular roles but also addresses key limitations of traditional protein–protein interaction analyses. By facilitating spatiotemporal investigations with exceptional accuracy, APEX is revolutionizing proteomics and molecular biology, unlocking new opportunities to explore dynamic biological systems and complex mechanistic questions.

## Figures and Tables

**Table 1:** Overview of substrates targeted by APEX for biochemical applications.

Substrates	Molecular Formula	CAS Registry Number	References
Hydrogen Peroxide	H_2_O_2_	7722-84-1	[62]
Ascorbic Acid	C_6_H_8_O_6_	50-81-7	[62]
Guaiacol	C_7_H_8_O_2_	90-05-1	[50,62,78]
Pyrogallol	C_6_H_6_O_3_	87-66-1	[62]
P-Cresol	C_7_H_8_O	106-44-5	[50,81]
o-Dianisidine	C_14_H_16_N_2_O_2_	119-90-4	[82]
Salicylhydroxamic acid	C_7_H_7_NO_3_	89-73-6	[50]
3,3’-Diaminobenzidine	C_12_H_14_N_4_	91-95-2	[83]
Amplex™ Red	C_14_H_11_NO_4_	119171-73-2	[12,84]
Biotin-tyramide / Biotin–phenol	C_18_H_25_N_3_O_3_S	41994-02-9	[70]
Biotin-LC-LC-tyramide (BxxP)	C_30_H_47_N_5_O_5_S	851113-28-5	[85]
Desthiobiotin-Phenol	C_18_H_27_N_3_O_3_	2242902-55-0	[86]
Adamantane-Phenol	C_24_H_36_N_2_O_4_	–	[87]
Biotin-Aniline	C_18_H_26_N_4_O_2_S	769933-15-5	[88]
Biotin-Naphthylamine	C_23_H_30_N_4_O_2_S	2375201-60-6	[88,89]
Alkyne tyramide/Alkyne–phenol	C_13_H_15_NO_2_	1694495-59-4	[90]

**Table 2:** Applications of APEX proximity labeling in biological research.

	Method/Strategy	Biological Question Addressed	Reference
Organelle Mapping	Mitochondria-targeted APEX-catalyzed proximity labeling	Comprehensive proteomic mapping of mitochondrial compartments	[70]
	Mapping Intermembrane space-selective APEX biotinylation	Profiling mitochondrial intermembrane space proteome	[103]
	APEX2-directed evolution for enhanced subcellular proteomics	Improved labeling efficiency for organelle-resolved proteomic studies	[14]
	APEX2 proximity proteomics in *Trypanosoma brucei* flagellum	Resolving proteome composition of flagellum subdomains	[97]
	APEX-based proximity labeling in *Plasmodium*	Identification of microneme proteins in *Plasmodium berghei* ookinetes	[148]
Protein-Protein Interactions	Multidimensional proximity labeling of GPCR complexes with APEX2	Tracking transient signaling networks	[108]
	APEX2-mediated proximity labeling in *Plasmodium falciparum* infected erythrocytes	Identification of KAHRP interactors	[102]
	CRISPR/Cas9 genome editing and APEX2	Study of native protein–protein interactions in live Drosophila ovary tissue	[121]
Protein-RNA Interactions	APEX-RIP (RNA Immunoprecipitation) approach	Mapping RNAs localized to specific proteins or compartments	[142]
	Proximity-CLIP combines APEX biotinylation with UV-induced cross-linking	Maps regulatory interactions within critical cellular compartments	
	APEX-Seq (APEX-catalyzed RNA biotinylation and sequencing)	Subcellular transcriptome profiling with spatial resolution	[136]
	dCas13-APEX2 by a guide RNA to target unmodified hTR	Tagging of endogenous proteins	[145]
	dCas13d-APEX2 fusion targeting FAS1 mRNA	Identification of mRNA-specific interacting proteins	[146]
	Spatially resolved proteomics using optimized APEX2	High-resolution mapping of cellular microdomains	[147]
Protein-DNA Interactions	APEX2 fusion targeting nuclear lamina and DNA-associated proteins	Mapping nuclear DNA–protein interaction landscapes	[100]
	dCas9-APEX2 adapted to DNA-associated proteins	Specific profiling of subnuclear proteomes of telomeres and centromeres	[140]
	Proximity labeling of mitochondrial DNA G-quadruplex interactors	Identification of G4-binding proteins in mitochondrial DNA	[141]

## Data Availability

Not applicable.
